# Effect of Electric Pulse Treatment on Hot-Dip Aluminizing Coating of Ductile Iron

**DOI:** 10.3390/ma14237219

**Published:** 2021-11-26

**Authors:** Jie Dong, Shouqian Yuan, Yongtao Sun, Shuangping Yang, Xiangdong Xing, Xiaomei He

**Affiliations:** 1 School of Metallurgical Engineering, Xi’an University of Architecture and Technology, Xi’an 710055, China; sq-yuan@163.com (S.Y.); yang_sping@163.com (S.Y.); hxmei1980@126.com (X.H.); 2 Shanxi Huaxiang Group Co., Ltd., Linfen 041600, China; hx.syt@huaxianggroup.cn

**Keywords:** electric pulse, ductile iron, hot-dip aluminizing, dynamics, corrosion resistance

## Abstract

In this paper, hot-dip aluminizing of ferrite nodular cast iron was carried out after treating liquid aluminum with different electrical pulse parameters. Compared with that of conventional hot-dip aluminizing, the coating structure of the treated sample did not change, the surface was smooth and continuous, and the solidification structure was more uniform. When high voltage and large capacitance were used to treat the liquid aluminum, the thickness and compactness of the coating surface layer increased. The thickness of the alloy layer decreased, and, the compactness and the micro hardness increased, so the electric pulse had a certain inhibition on the formation of the alloy layer. The growth kinetics of the alloy layer showed that the rate-time index decreased from 0.60 for the conventional sample to 0.38 for the electric pulse treated sample. The growth of the alloy layer was controlled by diffusion and interface reaction, but only by diffusion. The AC impedance and polarization curves of the coating showed that the corrosion resistance of hot-dip coating on nodular cast iron was improved by electric pulse treatment.

## 1. Introduction

Ferrite-based ductile iron has excellent performance and a low cost. However, when used in high-temperature environments, harsh atmospheres, or industrial corrosion conditions, it easily oxidizes, corrodes, and has a short service life, which greatly limited its application.

Hot-dip aluminum plating was a chemical treatment process for substrate protection. It was a long-lasting anticorrosion method for iron and steel in the 1940s and 1950s after hot-dip galvanizing [1,2]. With the decrease of global zinc resource and the increase of zinc price, hot-dip aluminizing of metal components may become an alternative method.

Hot-dip aluminum plating was a treatment method in which metal was immersed in molten aluminum for some time. The liquid aluminum and the metal substrate were mutually diffused to enhance the surface properties of the metal [3].

Hot-dip aluminizing of steel combined the corrosion resistance of aluminum with the strength of steel, making it more distinctive than steel hot-dip galvanizing and thermal spray paint. The corrosion resistance of hot-dip aluminized metal parts was better than that of galvanized ones in the regions containing SO_2_, H_2_S, CO_2_, NO_2,_ and other industrial gases as well as in the oceanic atmosphere, humid environment, and rainy areas. After hot-dip aluminized steel, the obtained surface layer made the steel excellent in corrosion resistance, and the transition alloy layer produced at the same time made the steel heat-resistant. However, when hot-dip aluminized parts were used in corrosion-resistant applications, the presence of a thicker transition alloy layer made parts prone to embrittlement during bending, which affected their performance. Therefore, on the premise of ensuring the quality of hot-dip aluminizing, that was, the bonding between the surface layer and the substrate was firm and there was no stripping, the surface was continuously plated without leakage, and then the corrosion resistance of the substrate was improved.

In past research, the objects of hot-dip aluminizing were steel and other alloy materials. Nodular cast iron was widely used in automotive exhaust pipe, water, gas pipe, petrochemical, and other occasions requiring higher corrosion resistance. To improve the service life of cast iron materials, the research on hot-dip aluminizing of ductile cast iron has important industrial application value.

Compared with that of steel, cast iron contained higher carbon and silicon, which prevented the infiltration of aluminum with higher melting point and higher activity. The graphite in cast iron also increased the difficulty of hot-dip aluminizing process [4,5], thus affecting the structure and morphology of the coating. People conducted in-depth research on the formation, phase composition, and influencing factors of the coating to produce a uniform and dense coating.

Considering that hot-dip aluminum plating was essentially a thin-layer solidification process, the electric pulse technology was tried to apply to hot-dip aluminum plating of cast iron materials.

The pulsed electric field affected on the liquid structure of the melt. The effect of the molten aluminum on the hot-dip aluminum coating of cast iron after the electric pulse inoculation treatment had not been reported. This article discussed the effect of pulsed electric field treatment of pure aluminum melt on the growth and structure and corrosion resistance of hot-dip aluminum coatings.

## 2. Materials and Methods

The material of the hot-dip aluminized sample was QT400-18 ductile iron, and the sample size was 25 × 10 × 1.5 mm.

QT400-18 spheroidal graphite cast iron plating samples needed to be polished off the surface oxide film, and prepared for hot plating after alkaline washing, and water washing, pickling, water washing, plating assistance, and drying. The alkaline washing adopted 80–90 °C 20% NaOH solution for 3–5 min, the acid washing adopted 15% HCl solution, the acid washing was at room temperature for 5 min, and the residue was removed by washing with water. The auxiliary plating agent was 90–95 °C 6% KF + 4% NaCl aqueous solution, and the auxiliary plating time was 5 min. Dry it in an electric thermostatic drying oven at 120 °C for 10 min.

The method of contrast experiment was used in this paper. Pure aluminum with purity of 99.9% was put into a graphite crucible, heated to 740 °C for melting, holding 5min. Then, conventional hot-dip aluminum plating (conventional sample) was carried out. Electric pulse treatment was performed on the pure molten aluminum. The treatment parameters were voltage 1100 V, frequency 0.88 Hz, capacitance 100 μF, and voltage 2600 V, frequency 0.88 Hz, capacitance 100 μF, 200 μF, 300 μF, and treatment time was the 60 s. After the electric pulse action stops, hot-dip aluminizing was carried out (referred to as treatment sample).

To facilitate the observation of the microstructure of the coating and the measurement of the thickness of the surface layer and the alloy layer, in the process of preparing the sample, a transverse section with a good coating and perpendicular to the surface should be selected. The sample was inlaid, and the inlay material was Bakelite powder.

The prepared samples were etched with 3–5% nitric acid alcohol after polishing. The microstructure and composition of the coating were observed by Olympus GX-51 inverted metallographic microscope (OLYMPUS, Tokyo, Japan), ZEISS Gemini SEM 300scanning electron microscope and energy dispersive spectrometer (Carl Zeiss, Oberkochen, Germany). The phase of the coating was analyzed by D8 ADVANCE A25 X-ray diffractometer (Bruker, Karlsruhe, Germany). The average thickness of the coating was obtained by multiple observations with an optical microscope.

The corrosion resistance of hot-dip aluminizing coating was tested. The working area of the electrode sample in the electrolyte was 1 cm^2^, and the sample was cleaned with acetone, ethanol, and distilled water. The corrosion solution was 3.5% NaCl solution. The reference electrode was calomel electrode, the auxiliary electrode was platinum electrode, and the sample was working electrode.

AC impedance measurement was carried out, and sine wave with the amplitude of 10 mv was applied at open circuit potential. The frequency range of measurement was 0.01 Hz–10,000 Hz, and the scanning speed was 20 MV/min. The polarization curve [6] was scanned by the potentiodynamic method in steady-state measurement. The scanning rate was 0.5 mv/s. The potential scanning range was ±200 mv (relative to the self-corrosion potential).

## 3. Results and Discussion

### 3.1. Microstructure and Structure of Hot-Dip Aluminium Coating

#### 3.1.1. Morphology of the Surface of Aluminized Coating

**Figure 1:**
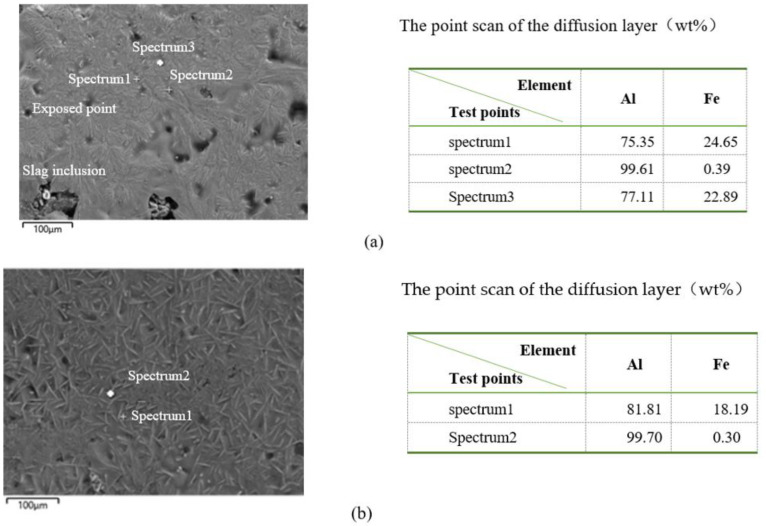
SEM images of surface of samples of hot-dip aluminized ductile iron. (**a**) Untreated; (**b**) treated.

**Figure 2:**
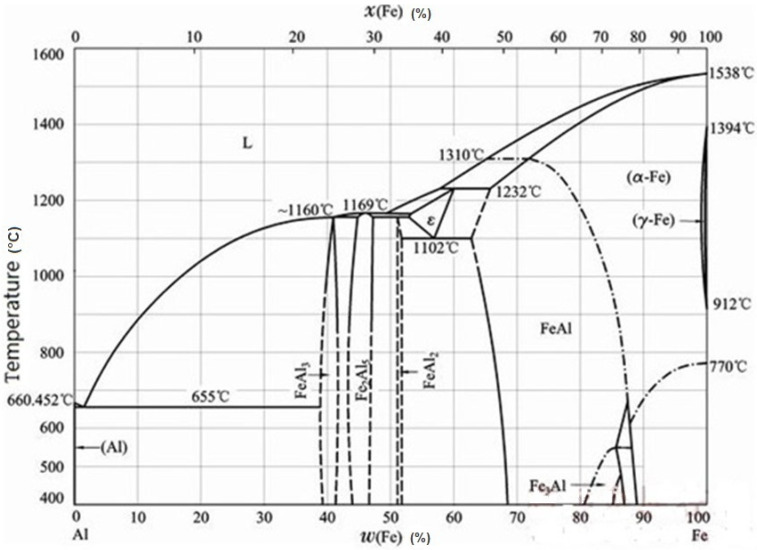
Fe-Al binary diagram.

The SEM images of the surface of conventional and treated samples of hot-dip aluminized ductile iron were shown in Figure 1. After hot-dip aluminizing, the surface of the conventional sample was uneven, the exposed points were many and dense, and there were a lot of oxide slag. It had a dark gray surface with a white needle-like phase. The results of energy spectrum analysis showed that the dark gray substrate contained almost 100% Al (Figure 1a), which could be determined as pure aluminum phase. The composition of white acicular phase were 77.37% Al and 22.63% Fe (conventional sample) and 81.81% Al and 18.19% Fe (treatment sample), which could be determined as FeAl_3_ phase. It fitted the phase diagram of Fe-Al [7]. (Figure 2). This was mainly due to the diffusion of iron from the matrix into the molten aluminum during hot-dip aluminum plating. FeAl_3_ was formed by the reaction of the precipitated iron with aluminum during the solidification of the outer layer of molten aluminum due to the low solid solubility of iron in the aluminum at room temperature (0.052%).

After electro-pulse treatment of liquid aluminum, the surface of the treated sample was relatively flat, continuous, bare point and crack and oxide slag less. Compared with that of the conventional samples, the morphology of the white dendrimer on the surface was changed, and the dendrimer morphology was broken, showing independent needle-like phase, and had no directivity. The analysis of energy spectrum showed that the needle-like phase was FeAl_3_. The results showed that the surface microstructure of the treated sample was more uniform after the electro-pulse treatment.

#### 3.1.2. Microstructure of Cross Section of Aluminized Coating

**Figure 3:**
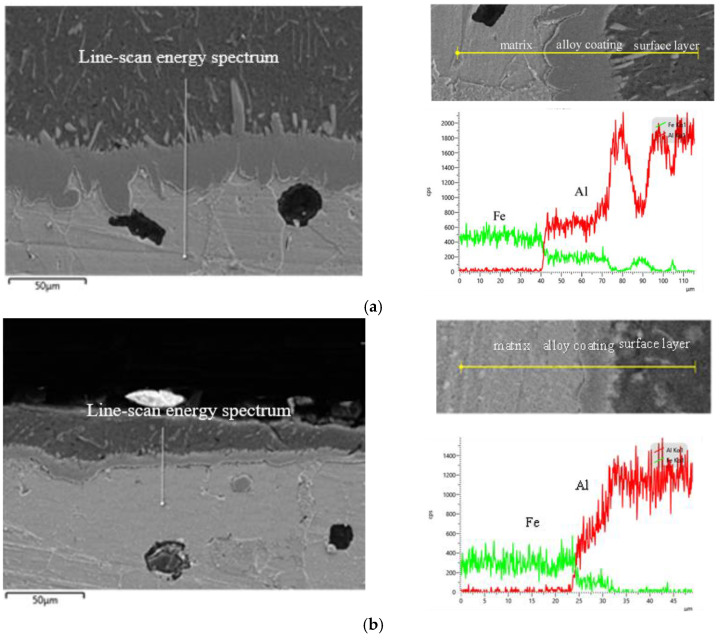
Line-scan energy spectrum of coating cross-section. (**a**) Untreated; (**b**) treated.

The cross-section morphology of hot-dip aluminized ductile cast iron as shown in Figure 3 could be seen that the aluminized layer outside the ductile cast iron consisted of two parts, the surface layer (the gray-black part) and the tongue-like substance diffusion layer extending to the substrate (the grey part). The line-scan energy spectrum of the coating cross-section was shown in Figure 3. As could be seen from the Figure 3, the surface layer was mainly Al element, and Fe-Al compound also existed in the surface layer. The content of Fe and Al in the surface layer of the conventional specimen (Figure 3a) was shown as wave line. The distribution of Fe and Al on the surface layer of the treated sample (Figure 3b) was more uniform and the Fe content was lower than that of the conventional sample. The main components of the tongue-like substance adjacent to the substrate were Fe and Al. The Fe elements were detected in the outer layer of ductile iron, which indicated that the elements were diffused from the matrix to the outer aluminum layer during immersion plating.

**Figure 4:**
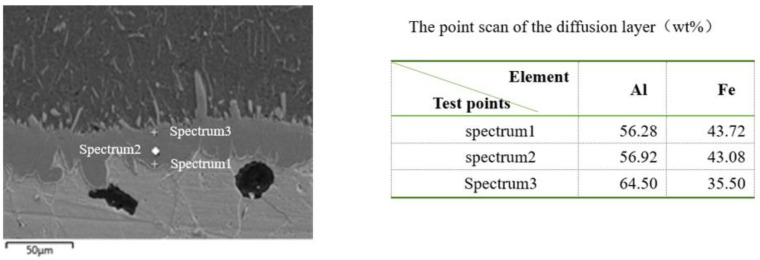
Point scans of diffusion layer.

According to study, during the hot-dip plating process of ductile iron, aluminum atoms, and iron atoms diffused each other [8], and a chemical reaction occurred at the phase boundary to form Fe-Al intermetallic compound. The compound then formed a tongue-like diffusion layer that extended to the substrate, that was, the alloy layer [9]. The point scan results of the diffusion layer were shown in the Figure 4. It was composed of AlFe_3_ and Al_2_Fe_5_. Therefore, the plating layer was composed of surface layer (aluminum-rich layer), iron-aluminum alloy layer (FeAl_3_ and Fe_2_Al_5_ iron-aluminum intermetallic compound) from the outside to the inside.

### 3.2. Effect of Electric Pulse Treatment on Surface Layer Thickness

**Figure 5:**
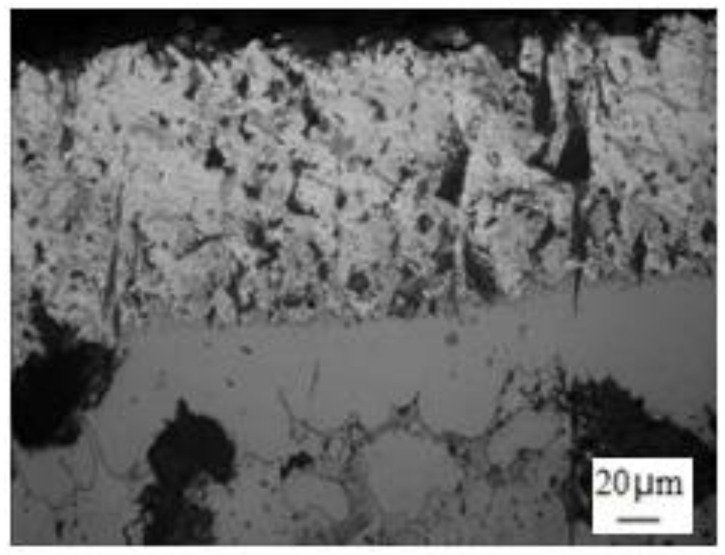
Microstructure of hot-dip aluminizing of ductile iron treated by electric pulse.

Figure 5 showed the cross-section of the hot-dip coating of molten aluminum treated by an electric pulse. The structure and organization of the aluminum-plated layer under the pulse current were the same as the conventional sample. It was also composed of a surface layer and an alloy layer.

**Figure 6:**
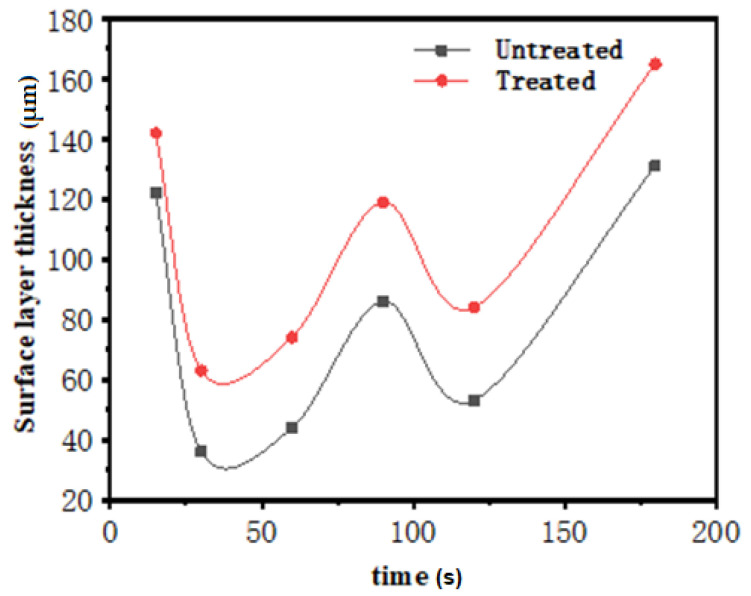
Variation of surface layer thickness before and after electrical pulse treatment.

Figure 6 showed the dynamic curve of the thickness of the aluminum surface layer over time in the two states of molten aluminum (electric pulse treatment and untreated). It showed that thickness variation of the two was the same trend. However, the surface layer of the hot-dip coating became thicker and denser after the molten aluminum was processed by an electric pulse.

At the initial stage of the immersion, because the surface temperature of the sample was low, it absorbed the heat of the aluminum liquid. It caused the plating liquid to solidify quickly on the surface of the sample to form a hard shell. With the increase of immersion time, the temperature of the immersion solution transferred to the solidified crust, which made the crust melt and its thickness decreased gradually. This was why the thickness of the surface layer decreased with time in the initial stage of immersion plating. With the hot-dip aluminizing, the atoms at the interface between the cast iron and aluminum liquid diffused each other. The Al-Fe intermetallic compound was formed at the interface of the matrix, and the aluminum rich layer containing iron was formed outside. Its melting point was higher, and the primary crystal nucleus might be formed at the immersion temperature, which increased the viscosity of the interface aluminum liquid and the thickness of the surface layer with time. Simultaneously, the core temperature of cast iron was lower than the interface temperature. When the initial solidified aluminum layer melted, the heat of the interface was transferred to the core of the cast iron, the interface temperature decreased, and the aluminum film layer thickened. Due to the interdiffusion of Fe and Al atoms at the interface, the iron content in the surface layer increased, the thickness of the surface layer decreases with the formation and growth of the alloy layer, and finally reached the diffusion equilibrium state. After that, the change of the surface layer thickness was only related to the viscosity of liquid aluminum, and it was little affected by temperature and time [10].

The iron-aluminum system had good wettability. The substrate processed by the auxiliary plating before the hot-dip aluminizing, so the aluminum liquid could be completely infiltrated with the substrate. Under the experimental conditions, the lifting velocity of the sample belonged to the stagnation range, so the surface state of the substrate had little effect on the velocity distribution. Therefore, the viscosity of the plating solution and the cooling rate of the specimen after leaving the plating solution also affected the thickness of the surface layer [11,12]. The greater the viscosity of the plating solution, the thicker the coating was, and the slower the plating solution fell when the sample left the plating solution, the thicker the surface layer would be formed.

The change of viscosity after the electric pulse treatment of molten aluminum had an important effect on the thickness of aluminum surface layer. The viscosity of metal melts depended largely on the movement of atoms in the liquid and the combination and bonding between them. The viscosity of molten metal changed exponentially with temperature, i.e., the Arrhenius function [13] was used to characterize the relationship between melt viscosity and temperature:

(1) $$\mu =\mathrm{Aexp}\left(\frac{\varepsilon }{\mathrm{kT}}\right)$$

where: $A=h/v_{m}$, h—Planck constant; $v_{m}$—Flow group (atoms, ions or clusters) size; μ—Viscosity; k—Boltzmann constant; ε—Viscous activation energy, is the activation energy required for the flow group to move from one equilibrium position to another equilibrium position; T—Absolute temperature.

Liquid aluminum melt was composed of single Al Atom and an atomic cluster Al-Al that satisfied a certain magic number [14]. The cluster structure which satisfied the magic number was more stable in size and higher in cohesive energy and was not a “short-range ordered” structure with time gathering and time dispersion. When the electric pulse applied to the melt, the electron migration reduced the static charge density in the outer layer of the cluster, and the electric field distortion reduced the potential of the outer layer on one side of the cluster. When the distortion reached a relaxed state, a certain number of individual atoms captured to form larger clusters that satisfied the next magic number. The pulsed output current also increased the collision frequency of atoms or clusters, making it easier to form large-scale clusters with the characteristic of the magic number. Therefore, the ions gathered inside the aluminum melt increased and were in a thermodynamically stable state. The value of $v_{m}$ reflected the size of the fluid cluster in the vessel. After the electric pulse treatment, the value of $v_{m}$ increased. Because of the strong binding force and viscous force between the clusters, and the large energy required to break the binding force, the ε value of the aluminum melt treated by electric pulse was slightly larger than that of the untreated that [15]. As a result, the viscosity of molten aluminum increased. The surface layer thickens, the ions accumulated in molten aluminum increase and the solidification structure becomes denser. As a result, the viscosity of molten aluminum increased after electric pulse treatment, and the surface layer of hot-dipped nodular cast iron became thicker. The solidification structure was more compact due to the increase of ions gathered in the flow group. Simultaneously, the electric pulse treatment had the effect on the growth of the alloy layer, so the change of the surface layer thickness would be different.

### 3.3. Effect of Electric Pulse Treatment on Alloy Coating Thickness

**Figure 7:**
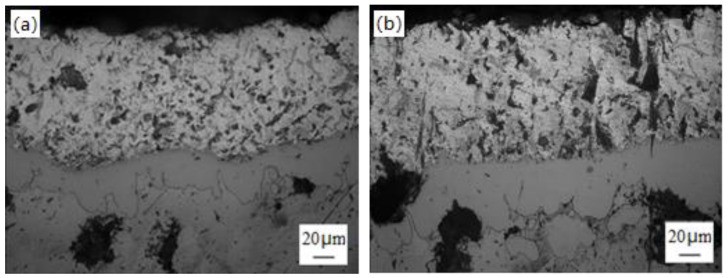
The morphology of coating at different dip time of hot-dip Aluninium plating without electric pulse. (**a**) 15 s; (**b**) 90 s.

**Figure 8:**
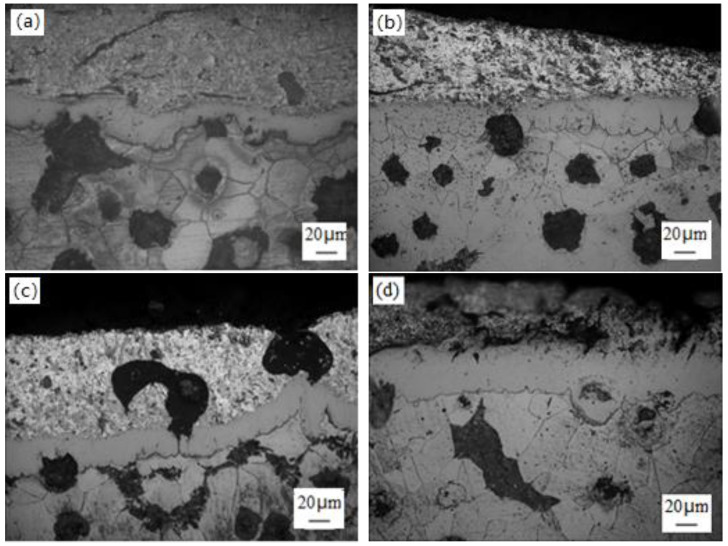
Morphology of aluminized layer with different capacitance parameters applied electric pulse. (**a**) Voltage 1100 V; capacitor 100 μF; immersion 90 s; (**b**) voltage 2600 V; capacitor 100 μF; immersion 90 s; (**c**) voltage 2600 V; capacitor 200 μF; immersion 90 s; (**d**) voltage 2600 V; capacitor 300 μF; immersion 90 s.

Figure 7 showed the morphology of the coating without electric pulse treatment. The alloy layer grew rapidly in 15 s time. However, the alloy layer grew very unevenly, and the bonding layer with the cast iron matrix appeared serrated, which indicated that the diffusion of iron and aluminum was fast. There was a discontinuity in the alloy layer. With the extension of the immersion plating time, the alloy layer became uniform. Figure 8 showed the coating morphology treated by different electric pulse parameters and the same immersion time. 

Compared with that of Figure 7b, the tooth shape of the diffusion layer of the conventional sample was irregular and discontinuous, and the height of the tooth peak was quite different. There were many independent tooth points in the front of diffusion layer, and the phenomenon of tooth bifurcation and oblique tooth existed. The front edge of diffusion layer of the treated sample was relatively flat, the tooth shape was continuous and regular, and the tooth roots were fused together. There were also bifurcation and helical tooth phenomena, but there were relatively few independent tooth points.

Compared with that of Figure 8b, the growth of the alloy layer was restrained, and the thickness of the alloy layer was reduced after the electric pulse was applied to the molten aluminum. With the increase of voltage and capacitance, the growth of the alloy layer gradually stabilized. When the voltage was 2600 V and the capacitance parameter was 300 μF, the plating alloy layer was thicker and uniformly continuous.

The time of electric pulse acting on aluminum liquid was the 60s, and the processing parameters were voltage 2600 V, capacitance 300 μF, and frequency 0.88 Hz. The immersion plating time of cast iron sample were the 60 s, 90 s, 120 s, 180 s, 210 s. The thickness of the alloy layer with different immersion plating time was investigated without and with electric pulse treatment.

**Figure 9:**
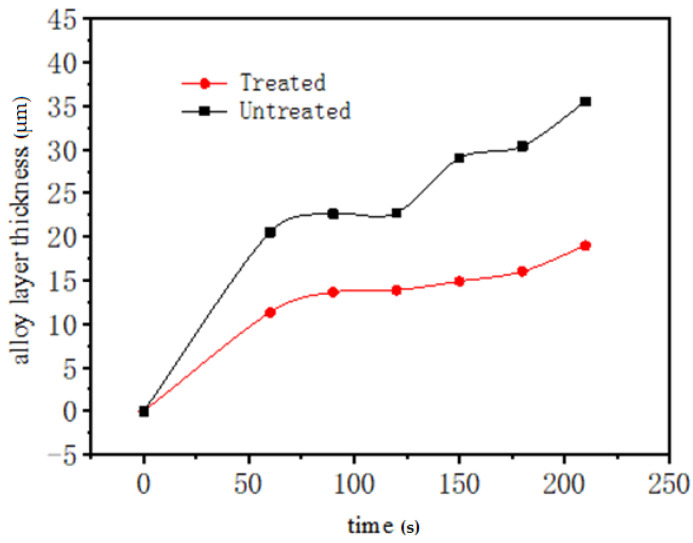
Thickness of aluminum alloy layer untreated and treated by pulse treatment.

The relationship between the thickness of the alloy layer and the time of immersion plating was shown in Figure 9. The thickness of the alloy layer increased rapidly with time in both cases. Within 90 s, the alloy layer changed violently. After 90 s, the rate of increase slowed down. Within 90 s, the growth of the alloy layer was influenced by the aluminum liquid treated by electric pulse, and the growth rate of the coating was slightly slow. The thickness of the alloy layer decreased by 19.5 μm compared with that of the sample without electric pulse treatment, which showed that the aluminum liquid treated by electric pulse could restrain the excessive growth of the alloy layer thickness.

### 3.4. Effect of Electric Pulse Treatment on the Growth of Alloy Layer

Hot-dip aluminum plating on metal surfaces was a complex surface physical and chemical process. The most important thing in the interface reaction process of hot-dip aluminum plating was diffusion. During hot-dip aluminizing of ductile iron, the iron in the matrix dissolved into molten aluminum, and the aluminum atoms diffused into the iron matrix to form intermetallic compounds. The process could be divided into two stages: the chemical reaction between iron and aluminum atoms to form a stable FeAl3 (or Fe_2_A1_5_) phase; the second was that aluminum and iron diffused through the formed phase layer and reacted to thicken the phase layer. In the immersion plating, the concentration and concentration gradient of Fe and Al varied with the reaction-diffusion process, so the process belonged to unsteady-state diffusion [16]. 

The empirical formula for the thickness and time of the alloy layer was [16]:

(2) $$d={\mathrm{kt}}^{n}$$

where D—alloy layer thickness (μm), t—reaction time (s), n—growth rate time index, k—growth rate constant.

The growth kinetics of the coating was calculated. When n = 0.5, the diffusion rate controlled the growth of the alloy layer, and the thickness of the alloy layer was parabola with time.

When n = 1.0, the controlling factor was the interface reaction rate during the growth of the coating, and the thickness of the alloy layer increased linearly with time.

The growth rate time index under different conditions was shown in Table 1.

Table 1 shows that the growth rate time index of the alloy layer of the conventional sample was 0.60, indicating that the growth of the alloy layer thickness was jointly controlled by the interface reaction rate and the diffusion rate.

For the sample treated by electric pulse, the growth rate index of the alloy layer was 0.38, indicating that the growth of the alloy layer was only controlled by diffusion, and the aluminum liquid treated by electric pulse has a certain inhibitory effect on diffusion.

During the hot-dip aluminum plating process, the growth rate of the alloy layer depends on the moving state of the alloy layer/metal matrix interface and alloy layer/molten aluminum interface. The alloy layer/metal matrix interface moved forwards, and diffusion and reaction occurred. The alloy layer/molten aluminum interface moved backward to dissolve, and the thickness of the alloy layer depended on the speed of the two.

At the beginning of immersion plating, the dissolution of iron and the adsorption of aluminum atoms on the iron surface proceed simultaneously, and an alloy layer began to form on the protruding parts of the iron surface. When the electric pulse was not applied, the lattice of the protruding part had larger distortion and high free surface energy, so the discontinuous alloy layer was formed. Due to the surface work hardening and the adsorption of aluminum during the processing of the workpiece, the initial temperature of iron recrystallisation was reduced, the iron recrystallisation speed was accelerated, and the recrystallisation tendency of the sample surface increased; the recrystallisation of the iron surface layer promoted the rapid formation and subsequent growth of new phase intermetallic compound crystals, and accelerated the growth rate of the alloy layer. The growth mode was controlled by the interface reaction and diffusion [17], but the nucleation work and diffusion activation energy of the new phase were higher, and nucleation and diffusion were relatively difficult. The pulse current changed the motion state of aluminum atoms, reduced the nucleation barrier and diffusion barrier, that is, reduced the nucleation work and diffusion activation energy [18], and promoted the diffusion of atoms, resulting in an increase in the nucleation rate. The phase interface increased, the atoms were arranged tightly, and diffusion was hindered. Therefore, the alloy layer grew slowly, and the thickness was reduced. That was, the growth of the alloy layer was only controlled by diffusion, as shown in Figure 9. Under the action of different electric pulse parameters, the micro hardness of the alloy layer also increased, and the microstructure of the alloy layer became dense. The test results were shown in Table 2.

### 3.5. Verification of Corrosion Resistance of Dip Coating

The surface layer obtained by hot-dip aluminizing of ductile iron should have excellent corrosion resistance, so the corrosion resistance of hot-dip aluminizing layer treated by electric pulse was tested. The electrochemical corrosion resistance of the coating in 3.5% NaCl solution was tested by CS series electrochemical workstation, and the AC impedance and polarization curves were measured.

**Figure 10:**
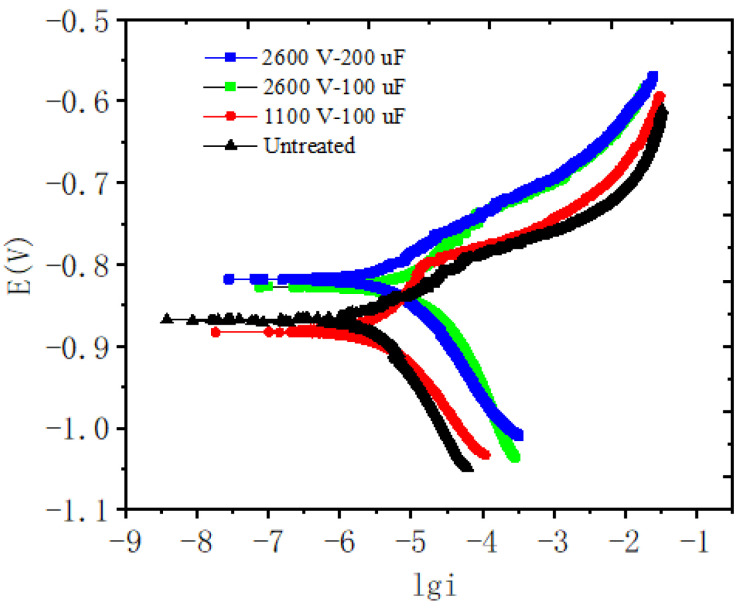
Potentiodynamic polarization curves of coating in 3.5% NaCl solution.

The potentiodynamic polarization curve of the hot-dip aluminum coating of ductile iron in 3.5% NaCl aqueous solution was shown in Figure 10. The corrosion current density and corrosion rate were shown in Table 3. With the increase of the electric pulse parameter voltage and capacitance, which treated aluminum liquid, the corrosion current density decreased, and the corrosion resistance of the coating increased.

**Figure 11:**
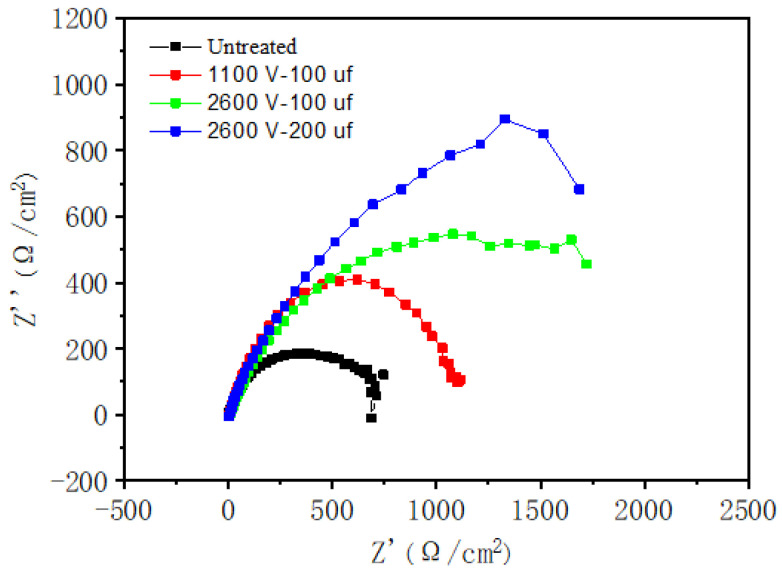
AC impedance spectrum of coating in 3.5% NaCl solution.

The AC impedance spectrum of the hot-dip aluminized samples in 3.5% NaCl aqueous solution was shown in Figure 11.

The results showed that the resistance value of the hot-dip aluminum coating after electric pulse treatment was greater than that without electric pulse treatment, indicating that the corrosion resistance of the hot-dip aluminum coating after electric pulse treatment was better than that of normal hot-dip. Moreover, with the increase of electric pulse parameter voltage and capacitance, the corrosion resistance of the coating increased.

Studies showed, in terms of the electrochemical properties of aluminum, that the electrode potential of aluminum was negative in most of the solutions. However, due to the formation of dense oxide film (Al_2_O_3_) or hydrated oxide film on aluminum in many cases, three kinds of corrosion, α- Al_2_O_3_, β- Al_2_O_3_·H_2_O and α- Al_2_O_3_·H_2_O, were often encountered in corrosion. When aluminum was passivated in the medium of PH 4~8, the solubility of the oxide film in the solution was small and the oxide film formed was stable [19,20]. Therefore, the corrosion resistance of aluminum plating was basically determined by the stability of the protective film on the surface of alumina in a given environment. 

**Figure 12:**
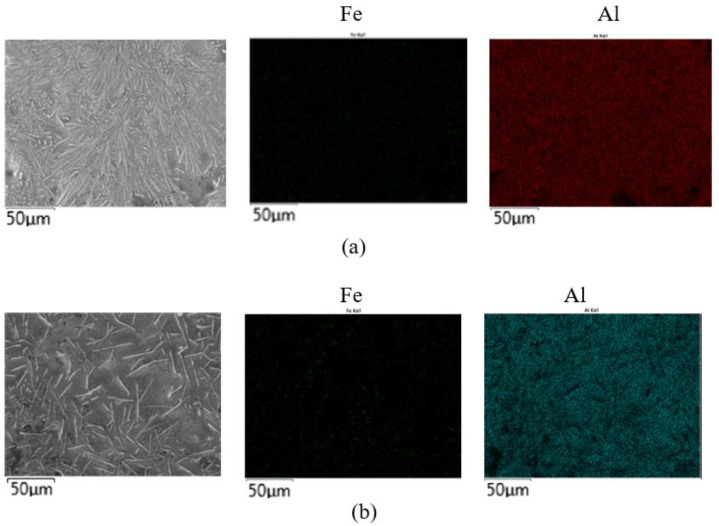
Surface layer scanning. (**a**) Untreated; (**b**) treated.

When the corrosive chloride anion existed in the corrosive medium, the oxidized metal ion Fe^+3^ could promote the pitting corrosion. When the content of Fe^+3^ in aluminum increased, the stability of the Al_2_O_3_ film nearby would be destroyed [20]. Due to the small radius of Cl^−^ ion, it was easy to invade the thin passivating film and produce the corrosion hole, and then the pitting corrosion occurred. Therefore, the iron content in the aluminum coating should be strictly controlled. The Figure 12 was the surface scanning. According to the surface scanning results (wt%) of the sample, the surface of the conventional sample contained 95.11% Al and 4.89% Fe, and the surface of the treated sample contained 96.43% Al and 3.57% Fe. The results showed that the iron content in the surface layer of the treated sample was lower than that in the conventional hot-dip aluminizing layer. The aluminum liquid was treated by electric pulse, the grain was refined and the surface structure was homogenized. Therefore, the corrosion resistance of hot-dip aluminizing coating was enhanced after electric pulse treatment.

## 4. Conclusions

After electric pulse treatment of molten aluminum, hot-dip aluminizing of nodular cast iron was carried out:

1. Compared with that of the conventional sample, the surface of the treated sample was smooth and continuous, and the Fe-Al alloy phase on the surface also changed from dendritic to independent and nondirectional needle shape, which made the solidification structure of the treated sample more uniform.
2. With higher voltage and larger capacitance, the surface layer of the coating was thickened, the alloy layer was thinner and the microhardness was increased, so the microstructure was denser. After electric pulse parameter voltage 2600 V, capacitance 300 uF, treatment of aluminum liquid 60 s, hot-dip aluminum plating time 90 s, the alloy layer was thinned by 19.5 μm. The electric pulse increased the viscosity of liquid aluminum and thickens the surface layer, but it inhibited the formation of alloy layer.
3. Considering the growth kinetics of hot-dip aluminum alloy coating, the growth rate time index of the conventional alloy layer was 0.60, which showed that the thickness of the alloy layer was controlled by the interface reaction rate and diffusion rate. The growth rate time index of the treated alloy layer was 0.38, which showed that the growth of the alloy layer was only controlled by diffusion.
4. The Fe content on the surface of the treated sample was reduced and the distribution was more uniform, so the corrosion resistance of the hot-dip coating of ductile iron was improved after electrical pulse treatment.

## Figures and Tables

**Table 1:** Growth kinetics parameters of hot-dip aluminum alloy layer.

Experimental Condition (Molten Aluminum)	Growth Rate Constant	Growth Rate Time Index
Untreated	1.51	0.60
Treated	2.39	0.38

**Table 2:** Micro hardness of alloy layer under different conditions.

Voltage (V)	Capacitance (μF)	Micro Hardness (HV)
0	0	325.8
1100	100	464
2600	100	512.87
2600	200	560.87
2600	300	570.9

**Table 3:** Corrosion current density and corrosion rate of coatings under different conditions.

Aluminum Liquid	Untreated	1100 V-100 μF	2600 V-100 μF	2600 V-200 μF
Corrosion current density/mA/cm^2^	0.0106	4.03 × 10^−3^	2.73 × 10^−3^	2.43 × 10^−3^
Corrosion rate/mm/a	0.1153	4.39 × 10^−2^	2.97 × 10^−2^	2.65 × 10^−2^

## Data Availability

Data is contained within the article.

## References

[1] Sun J., Wan M. (2010). Compartion of corrosion resisting properties of hot dip garvanizing and aluminium coating of steel parts and their application. Mod. Mach..

[2] Shi Z., Cao J., Han F. (2014). Preparation and characterization of Fe-Al intermetallic layer on the surface of T91 heat resistant steel. J. Nucl. Mater..

[3] Lemmens B., Springer H., Graeve I.D., de Strycker J., Raabe D., Verbeken K. (2017). Effect of silicon on the microstructure and growth kinetics of intermetallic phases formed during hot dip aluminzing of ferritic steel. Surf. Coat. Technol..

[4] Lin X., He Y., Jiang Y. (2005). Research progress in aluminizing process of cast iron. Mater. Sci. Eng. Powder Metall..

[5] Zhong F., Zhou J. (1991). Utilizing of Iron Castings. Castings.

[6] Hu H., Li N. (2007). Electrochemical Measurement.

[7] Bouche K., Barbier F., Coulet A. (1998). Intermetallic compound layer growth between solid iron and molten aluminium. Mater. Sci. Eng..

[8] Shi X., Meng J. (2014). Research on diffusion behaviour of hot-dipped aluminium coating on ductile iron. J. Heilongjiang Univ. Sci. Technol..

[9] Jia J., Zhu W., Xiong W., Zhou J. (2014). Influence of diffusion ways on morphology of plating layers of hot dip aluminizing stainless steel sample. Trans. Mater. Heat Treat..

[10] Yuan X., Mei Y., Ping Z.R. (1996). The affecting of temperature and time to the growth of hot-dip aluminizing coating on A3. Trans. Nonferrous Met. Soc. China.

[11] Liu S., Song S., Gao H. (2000). Study on continuous hot-dip aluminum plating for steel wire. Mater. Prot..

[12] Song S., Liu S., Li C. (2000). Continuous hot-dip aluminium plating for steel wire. Heat Treat. Met..

[13] Suzuka R., Nishimura M. (2002). Variation of silicon melt viscosity with boron addition. J. Cryst. Growth.

[14] Turnbull D. (1952). Kinetics of solidification of supercooled liquid mercury droplets. Chem. Phys..

[15] Wang J., Qi J. (2011). Theory and Application of Metal Melt Electric Pulse Treatment.

[16] Liu B. (1995). Hot-Dip Aluminizing of Steel.

[17] Qi Z. (1998). Diffusion and Phase Transformation in Solid Metal.

[18] Tang Y. (2000). Investigation of Liquid Metals Structure and Steel Solidification Improvement under Various Electropulse Conditions. Ph.D. Thesis.

[19] Wang Y., Zhang N. (1991). X-ray studies of Al/A1_2_O_3_ multilayered films. Mater. Sci. Eng..

[20] Yang S., Chang T. (2003). Material Corrosion and Protection.

